# Elicitation of Protective Immune Responses Against Influenza Virus Following Intranasal Delivery of Fluzone or Flublok Vaccines

**DOI:** 10.3390/vaccines14010103

**Published:** 2026-01-21

**Authors:** Naoko Uno, Matthew H. Thomas, Camila Caetano, Ted M. Ross

**Affiliations:** 1 Center for Vaccines and Immunology, University of Georgia, Athens, GA 30605, USA; nuno@uga.edu; 2 Florida Research and Innovation Center, Cleveland Clinic, Port Saint Lucie, FL 34987, USA; thomasm36@ccf.org; 3 Department of Infection Biology, Cleveland Clinic Research, Cleveland, OH 44106, USA; caetanc@ccf.org; 4 Department of Infectious Diseases, University of Georgia, Athens, GA 30602, USA

**Keywords:** influenza, vaccination, antibody, split-inactivated, recombinant HA

## Abstract

**Background/Objectives:** While new vaccines are in development; one strategy to increase influenza vaccine coverage is to repurpose current influenza vaccines for intranasal delivery. **Methods:** To address this goal; mice were vaccinated intranasally with either a split inactivated virus vaccine (Fluzone) or a recombinant HA vaccine (Flublok) at one of two doses (1 μg high dose or 0.1 μg low dose). Both vaccines were adjuvanted with either a STING agonist; c-di-AMP (CDA); or a combination of a synthetic toll-like receptor (TLR) 4 and TLR7/8 agonist (TRAC478). **Results:** Mice vaccinated with either vaccine plus adjuvant had higher hemagglutination-inhibition titers than mice administered unadjuvanted vaccines. Mice vaccinated with either vaccine plus CDA had on average higher numbers of H3 and influenza B hemagglutinin (HA)-specific antibody-secreting cells (ASCs); whereas mice vaccinated with vaccine plus TRAC478 had on average higher number of H1 HA-specific ASCs. All vaccinated mice challenged with the H1N1 influenza virus were protected against both morbidity and mortality with no detectable virus in their lungs. Mice challenged with the H3N2 influenza virus all lost weight over the first 5 days of infection. Adding TRAC478 with either a high or low dose vaccine resulted in 80–100% survival following challenge. Almost all mice vaccinated with Flublok plus CDA died from H3N2 influenza virus challenged with ~2 logs higher viral lung titers than mice administered Flublok only or Flublok plus TRAC478. **Conclusions:** Overall; Fluzone and Flublok can effectively be used for intranasal vaccination.

## 1. Introduction

The World Health Organization (WHO) estimates that influenza viruses cause significant morbidity and mortality, particularly among vulnerable populations, such as the elderly, young children, pregnant women, and individuals with chronic medical conditions, including metabolic syndrome or those with immunocompromising conditions [1,2]. Vaccination is one of the primary tools for combating influenza virus infection and disease. However, existing vaccines have limitations regarding their effectiveness and the level of protection. Since influenza viruses evolve rapidly, this leads to a decrease in the effectiveness of current vaccines, particularly when the circulating strains are mismatched to the vaccine components [2,3].

Current influenza vaccines primarily target the surface glycoprotein hemagglutinin (HA), since the elicitation of protective antibodies against HA is crucial to neutralize virus infection [3]. Several commercial seasonal inactivated influenza vaccines (IIV) that contain H1N1, H3N2, and B influenza strains are on the market [4]. Fluzone Standard Dose (SD), produced by Sanofi Pasteur, is a split inactivated virus vaccine and is the most commonly administered vaccine each season [5]. This egg-based vaccine contains 15 μg of HA per strain without the inclusion of an adjuvant [6,7]. Flublok, also produced by Sanofi Pasteur, is a recombinant HA-based influenza vaccine containing strains from the same three circulating subtypes [8,9]. Both these vaccines are administered intramuscularly and elicit robust neutralizing antibody responses against the HA of the matched virus [10].

In this study, these commercial influenza vaccines were administered intranasally to vaccinate mice. To enhance the effectiveness, each vaccine was administered in combination with one of two adjuvants. The first adjuvant, TRAC478, is a combination of two synthetic toll-like receptor (TLR) agonists, INI-2002, a synthetic TLR4 agonist, and INI-4001, a novel lipidated oxoadenine and TLR7/8 agonist. Both agonists signal through the TIR domain-containing adapter-inducing interferon-β (TRIF) and myeloid differentiation factor 88 (MyD88) pathways, driving the production of TNF-α and IL-6, ultimately enhancing Th1-biased B and T cell responses [11]. The second adjuvant administered with the Fluzone and Flublok commercial vaccines was bis-(3,5)-cyclic dimeric adenosine monophosphate (c-di-AMP or CDA) that is a stimulator of interferon gene (STING) activator and elicits strong mucosal, as well as systemic immune responses [12]. Intranasal vaccination with CDA provides cellular and humoral immunity with balanced Th1/Th2/Th17 responses [12] and a diverse antigen-specific IgG subclass profile [13]. In this study, mice were assessed for protective immune responses against H1N1, H3N2, and B influenza viruses following intranasal vaccination with Fluzone or Flublok mixed with adjuvant.

## 2. Materials and Methods

### 2.1. Propagation of Influenza Viruses

Influenza viruses used in this study were obtained from Virapur (San Diego, CA, USA) or generated from seed stocks provided by the Center for Disease Control and Prevention (CDC, Atlanta, GA, USA), Influenza Reagent Resources (IRR), or BEI Resources (BEI, Manassas, VA, USA). All virus seed stocks were expanded via a single passage in specific-pathogen-free embryonated chicken eggs. Single-use aliquots of each virus stock were stored at −80 °C. H1N1 influenza viruses used in this study include A/Brisbane/02/2018 (BR/18), A/Victoria/2570/2019 (VC/19), and A/Victoria/4897/2022 (VC/22). The H3N2 influenza viruses used in this study include A/Switzerland/9715293/2013 (SW/13); A/Massachusetts/18/2022 (MA/22); and A/District of Columbia/27/2023 (DC/23). The influenza B viruses (IBV) used for this study include B/Phuket/3073/2013, B/Washington/02/2019 (WA/19) and B/Austria/1359417/2021 (AU/21). The IBV viruses used for HAI assays were first ether-extracted to increase HA activity [14].

### 2.2. Vaccination and Infection of Mice

All animal procedures were approved by the Institutional Animal Care and Use Committee (IACUC). A total of 330 DBA/2J (female, 6 to 8-week-old) mice were purchased from Jackson Laboratories (Bar Harbor, ME, USA) and housed in microisolation cages with ad libitum access to feed and water. Animals were given a week to acclimate. All animal procedures were approved by the Cleveland Clinic Institutional Animal Care and Use Committee (IACUC approval #2935) and the University of Georgia (IACUC approval #A2024 06-001-Y1-A1). Mice were randomly divided using a random order generator into 11 vaccine groups (*n* = 30), and each group was further divided into two different viral challenge groups (*n* = 15). The sample size was chosen based on previous studies [15]. No inclusion/exclusion criteria were set, and no mice were excluded from the analyses. To minimize cofounders, mice were randomly chosen for spleen and lung collection before starting vaccination and infection. Researchers were blinded to the vaccine groups by assigning numbers instead of vaccine names to each group. Then mice were vaccinated intranasally with 1 or 0.1 μg of one of two commercial influenza vaccines, Fluzone or Flublok (Sanofi Pasteur, Swiftwater, PA, USA), without an adjuvant or formulated with either TRAC478 (Inimmune, Missoula, MT, USA) at a 1:1 ratio with vaccine or with 5 µg of c-di-AMP (Sigma, St. Louis, MO, USA). For mock-vaccination, mice were intranasally inoculated in 25 µL sterile 1× PBS. Mice were boosted with the same vaccine and adjuvant combinations on day 28. Blood samples were collected on day 14, 42, 49, and spleens (*n* = 3) were collected on day 37. On day 56, mice were challenged with either BR/18 (H1N1; 3.6 × 10^4^ PFU/mouse) or SW/13 (mouse-adapted H3N2; 5 × 10^6^ PFU/mouse) in 50 μL volume. Mice were weighed and monitored for signs of morbidity and mortality for 14 days post-challenge. Cumulative clinical scores (CS) were reported for each animal based on the following criteria: no clinical signs of disease (CS = 0); weight loss between 15% and 25% or lethargy (CS = 1); dyspnea (CS = 2); weight loss of 25% or greater, severe respiratory distress, or failure to respond to external stimuli (CS = 3). Lungs were collected at day 3 post-challenge. Any mice that reached clinical endpoint (CS ≥ 3) were humanely euthanized. Mice were anesthetized with 20% isoflourane until unconscious and euthanized with 5% CO2 by inhalation for 3 min and then treated to cervical dislocation. Then, a lack of heartbeat was verified manually.

### 2.3. Tissue Sampling

Whole blood was collected from the submandibular vein into BD Microtainer SST tubes and allowed to rest at RT for ≥30 min prior to serum separation or short-term storage at 4 °C. Serum separation was performed by centrifuging the whole blood samples at 10,000 rcf for 2 min. The total volume of sera was collected above the clot activator gel and transferred into 1.5 mL microcentrifuge tubes for long-term storage at −20 °C. Lungs were collected from 3 mice/vaccine group at 3 days post-challenge (dpc). To determine virus titer, lungs were collected using aseptic technique and placed in 1.5 mL microcentrifuge tubes on dry ice, then snap-frozen prior to transfer to a −80 °C freezer for long-term storage. Spleens and bronchial lavages were collected on day 39 (*n* = 3), 9 days post boost. Spleens were harvested in B cell media (1 L RPMI supplemented with 100 mL FBS, 20 mL HEPES, 20 mL of a 50× MEMAA solution, 10 mL of a 100× NEAA solution, 10 mL Penicillin-Streptomycin, 10 mL sodium pyruvate, 2 g of sodium bicarbonate, and 3.5 μL of 2-mercaptoethanol). Splenocytes were processed and stored in liquid nitrogen in freezing media (FBS with 1% DMSO).

### 2.4. Enzyme-Linked Immunosorbent Assay (ELISA)

ELISA was used to confirm immunogenicity of the vaccine +/− adjuvant, as well as to measure anti-HA IgG titers against the HA vaccine antigens from vaccinated mice. Immulon 4HBX 96-well plates (Thermo Fisher, Waltham, MN, USA) were coated with 100 µL/well at 1 µg/mL of either VC/22 H1 rHA, TH/22 H3 rHA, or AU/21 IBV rHA protein in Carbonate Coating Buffer (pH 9.4) as previously described [15,16]. Serum samples were two-fold serially diluted in Blocking Buffer and 100 µL of diluted serum was transferred to the ELISA plates. Plates were incubated in a humidified chamber at 37 °C for 2 h and then washed (5×) with 150 µL of wash buffer. Then, 100 µL of the appropriate detection antibody for IgG (goat α-mouse IgG, Southern Biotech, Birmingham, AL, USA) was added at a 1:4000 dilution and then incubated at 37 °C for 1.5–2 h. H_2_O_2_ was added containing 1 mg/mL of ABTS substrate to the plates at 100 µL/well. Plates were incubated with substrate for 15 min before the addition of 50 µL of 1% Sodium Dodecyl sulfate (SDS). Optical density in the wells was measured at 414 nm on an Epoch 2 microplate reader (BioTek, Winooski, VT, USA).

### 2.5. Hemagglutination Inhibition Assay

The HAI assay was used to evaluate HA-directed serum antibodies elicited by vaccination using a protocol adapted from the World Health Organization (WHO) Manual for the Laboratory Diagnosis and Virological Surveillance of Influenza [17]. Serum samples used for HAI assays were combined 1:3 with Receptor Destroying Enzyme (RDE; Denka Seiken, Tokyo, Japan) and incubated at 37 °C for 12–18 h to remove nonspecific inhibitors. The RDE was deactivated by heating the samples to 56 °C for 30–45 min. 1xPBS was added to the samples at room temperature to achieve a final serum dilution of 1:10. For the assay, 25 µL of each RDE-treated serum sample was 2-fold serially diluted across 96-well, V-bottom microtiter plates. An equal volume of virus tittered to 8 hemagglutination units (HAU)/50 µL was added to the samples in the microtiter plates. The H1N1 influenza virus strains were incubated with serum for 20 min and then mixed with 50 µL/well of 0.8% turkey red blood cells (RBCs; Lampire Biologicals, Pipersville, PA, USA) and incubated for 30 min at RT. The H3N2 strains were incubated with serum for 30 min, then 50 μL/well of 0.8% guinea pig RBCs (Lampire Biologicals, Pipersville, PA, USA) supplemented with 20 nM oseltamivir were added for a 60 min incubation at room temperature. After incubation with either RBC type, plates were tilted forward to identify the highest serum dilution in which RBCs did not agglutinate for each sample. The HAI titer is defined as the reciprocal of the highest dilution that prevents hemagglutination.

Vaccine-induced HAI titers for all groups were determined by measuring HAI titers in sera collected 2 weeks following the final vaccination against H1N1 strains: A/Victoria/2570/2019 (VC/19) and A/Victoria/4897/2022 (VC/22), H3N2 strains: A/Massachusetts/18/2022 (MA/22) and A/District of Columbia/27/2023 (DC/23), and against IBV strains: B/Washington/02/2019 (B/WA/19) and B/Austria/1359417/2021 (B/AU/21).

### 2.6. Enzyme-Linked Immunospot Assays

*Synthetic Peptides.* The 14 and 15-mer peptides from Influenza A/New York/18/2009 (H1N1) pdm09 (Catalog No. NR-19245), Influenza Virus A/Perth/16/2009 (H3N2) (Catalog No. NR-19266), and B/Malaysia/2506/2004 (IBV) (Catalog No. NR-18967) were obtained from BEI Resources Repository, NIAID, NIH. The 70-peptide array overlapping by 7 amino acids spans the whole hemagglutinin (HA) protein of the strains A/New York/18/2009 (H1N1) pdm09 (GenPept: ACU13097) A/Perth/16/2009 (H3N2) (GenPept: ACS71642.1), and B/Malaysia/2506/2004 (GenPept: ABU99194.1).

*Enzyme-linked cytokine immunospot assay.* T-cell enzyme-linked immunospot (ELISpot) assays were performed using ELISPOT Kit from Cellular Technology Limited (CTL) (Shaker Heights, OH, USA—mIFN-γ/IL4-1M/10 or IL-17) according to the manufacturer recommendations. Each spot corresponds to an individual cytokine-secreting cell. Background responses from negative control wells were subtracted to normalize the data across all experimental groups.

*B-cell ELISpot assay.* IgA/IgG Double-Color ELISPOT kit from CTL was performed following the manufacturer’s instructions and as previously described according to the manufacturer’s recommendations. Each spot corresponds to an individual antibody-secreting cell. Background responses from negative control wells were subtracted to normalize the data across all experimental groups.

### 2.7. Detection of Viral Lung Titers by Plaque Assay

Virus titers in the lungs of infected mice were evaluated at 3 days post-challenge via the viral plaque assay as previously described. The number of plaques in each well was determined, and the virus titers for each sample were defined as the number of plaque-forming units (PFU)/g of lung homogenate.

### 2.8. Statistical Analysis

All data are presented as mean ± standard error of the mean (SEM). One-way ANOVA was used to analyze lung virus titers after influenza viral challenge and to analyze ELISPOT counts between vaccine groups. Two-way ANOVA was used to analyze the total IgG antibody titers for each of the vaccine antigens between all vaccine groups and to analyze influenza strain-specific HAI titers between vaccination groups. HAI and IgA titers were transformed to log_2_ scale to standardize wide antibody range. Statistical analysis was performed using GraphPad Prism 9 software (GraphPad, San Diego, CA, USA). A “*p*” value less than 0.05 was defined as statistically significant (*, *p* < 0.05; **, *p*  <  0.01; ***, *p*  <  0.001; ****, *p* < 0.0001).

## 3. Results

### 3.1. Vaccinations and Infections

**Figure 1:**
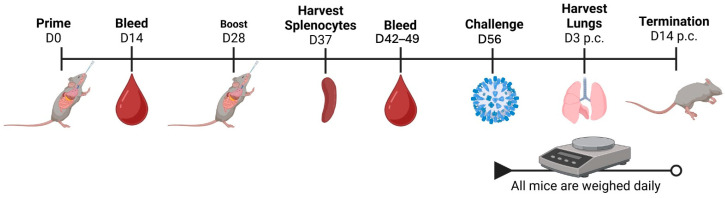
**Study regimen.** Influenza-naïve mice (A) were intranasally vaccinated (*n* = 15/group) on days 0 and 28, and bled on days 14, 42, and 49. Spleens were collected on day 37. Mice were then challenged on day 56 with BR/18 H1N1 influenza virus or the mouse-adapted SW/13 H3N2 influenza virus. Lungs were collected 3 days post-infection. Daily weights and clinical signs were recorded following either infection.

Mice (DBA/2J; *n* = 15, 6–8 weeks of age), were vaccinated with one of two commercial influenza virus vaccines at day 0 and boosted at day 28 (Figure 1). Mice were intranasally administered 1 μg (high dose) or 0.1 μg (low dose) of Fluzone Standard Dose or 1 μg (high dose) or 0.1 μg (low dose) of Flublok both manufactured by Sanofi Pasteur (Swiftwater, PA, USA). Each vaccine was formulated with one of two adjuvants, TRAC478 (Inimmune, Missoula, MT, USA) or c-di-AMP (CDA) (Invitrogen, Waltham, MA, USA) in a 1:1 ratio in a 50 μL solution. A set of mice were administered each influenza virus vaccine without adjuvant as a control. Mock vaccinated animals received adjuvant only in 0.9% buffered saline (Figure 1). Blood was collected from each mouse on days 0, 14, 42, 49, post-vaccination and spleens and BALF/lungs (*n* = 4) were collected on day 37 post-vaccination. On day 56, all mice were infected with one of two influenza viruses, A/Brisbane/02/2018 (1 × 10^4^ PFU/mouse) or A/Switzerland/9715293/2013 (5 × 10^6^ PFU/mouse). Lungs were collected from mice (*n* = 3) on day 3 post-infection and the remaining mice were monitored for signs of morbidity and mortality for 14 days post-infection.

### 3.2. Serological Assessment

**Figure 2:**
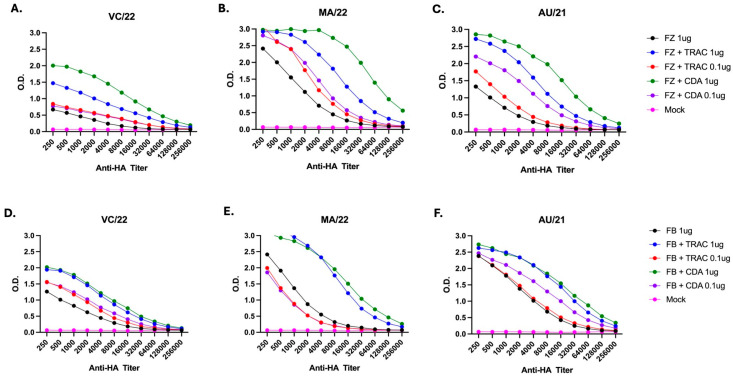
**Mouse total IgG ELISA titers.** ELISAs detecting the presence of total anti-HA IgG were performed using serum collected from each mouse post-boost and were pooled for each group. Plates were coated with either VC/22 (**A**,**D**) H1 rHA, MA/22 (**B**,**E**) H3 rHA, or AU/21 (**C**,**F**) IBV rHA and probed with different secondary antibodies specific to total IgG. Endpoint dilution titers are plotted on the y-axis and the vaccine groups.

All vaccinated mice had anti-HA antibodies that bound to rHA proteins representing the H1, H3, and B HA proteins in the vaccine (Figure 2). Mice vaccinated with a high dose of Fluzone or Flublok mixed with either adjuvant had the highest anti-HA antibody titers against all three vaccine components compared to unadjuvanted vaccine or low dose vaccine mixed with either adjuvant (Figure 2A–C). Mice vaccinated with high dose Fluzone plus CDA had higher anti-HA titers than mice vaccinated with the same high dose Fluzone mixed with TRAC478. In contrast, mice vaccinated with Flublok mixed with either adjuvant had the same anti-HA titers that were higher than low dose or unadjuvanted Flublok vaccine (Figure 2D–F).

**Figure 3:**
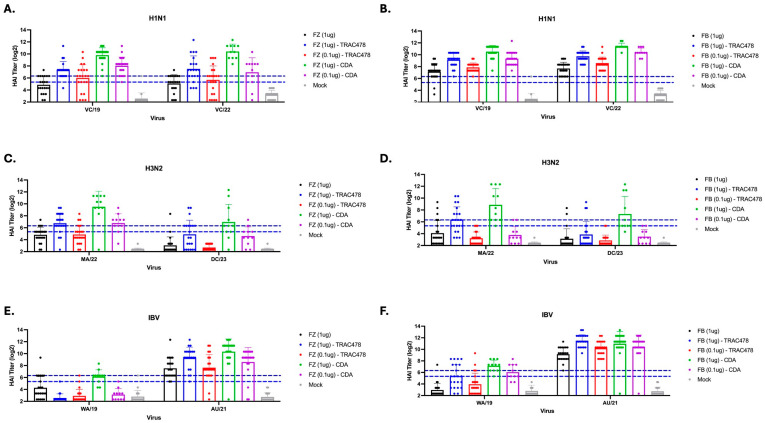
**HAI serum antibody titers induced by adjuvanted and non-adjuvanted 2024–2025 Fluzone or Flublok seasonal influenza vaccination.** HAI titers were assessed against two H1N1 viruses (**A**,**B**), two H3N2 viruses (**C**,**D**), and two influenza B viruses (**E**,**F**). Log_2_ HAI titers for individual mice are shown as scatter dot plots with bars representing the mean titer of each group and error bars indicating the standard deviation. Dotted lines indicate a 1:40 to 1:80 HAI titer range. Statistical analyses were performed using one-way ANOVA to compare the means of each column.

Mice vaccinated with a high dose of Fluzone mixed with TRAC478 had on average antibodies with HAI activity between 1:160 and 1:320 against the H1N1 influenza viruses (Figure 3A). These HAI titers were significantly higher than titers in mice vaccinated with a low dose of Fluzone mixed with TRAC478 with the average titer ranging between 1:40 and 1:80 against the H1N1 influenza viruses. Mice vaccinated with unadjuvanted Fluzone (1 mg) had similar HAI titers as mice vaccinated with the low dose of Fluzone plus TRAC478 (Figure 3A). Mice vaccinated with a high dose of Fluzone mixed with CDA had roughly 3-fold higher HAI titers against the H1N1 influenza viruses compared to mice vaccinated with the equivalent Fluzone dose plus TRAC478 (Figure 3A). In addition, mice vaccinated with low dose Fluzone plus CDA adjuvant had on average HAI titers ≥ 1:40 against the H1N1 influenza viruses.

All mice vaccinated with Flublok, with or without adjuvant, had HAI titers ≥ 1:80 against the H1N1 influenza viruses (Figure 3B). The addition of either TRAC478 or CDA adjuvant mixed with a high dose of Flublok had HAI titers that were, on average, 2–4 fold higher than mice vaccinated with Flublok only against the H1N1 influenza viruses (Figure 3B). Mice vaccinated with the low or dose of Flublok mixed with CDA had 1- or 2-fold higher HAI titers than mice vaccinated with equivalent doses of Flublok formulated with TRAC478.

Mice vaccinated with unadjuvanted Fluzone or Flublok had in general low to undetectable titers against the H3N2 influenza viruses (Figure 3C,D). Mice vaccinated with either high dose of Fluzone and Flublok and mixed either adjuvant had on average 1:320–1:640 HAI titers against the H3N2 influenza virus strain MA/22 and average HA titers against DC/23 of ~1:160 (Figure 3C,D). A few mice had high HAI titers against DC/23; however, most mice had low to undetectable titers. Mice vaccinated with Fluzone plus CDA had average HAI titers ~1:160 against DC/23 (Figure 3C). Few mice vaccinated with low dose vaccine had HAI titers ≥ 1:40.

Mice vaccinated with Fluzone plus TRAC478 had high HAI titers against the vaccine matched B/AU/21 virus on average 1:2084–1:4096 (Figure 3E) and were statistically similar to mice vaccinated with high dose Flublok plus CDA (Figure 3F). Mice vaccinated with a low dose of Fluzone mixed with either adjuvant had average HAI titers against B/AU/21 that were 2–4-fold lower than mice administered high dose vaccine plus adjuvants and these titers were similar to HAI titers in mice administered unadjuvanted vaccine. Mice vaccinated with high dose of Fluzone mixed with TRAC478 had average HAI titers between 1:40 and 1:80 against B/WA/19 (Figure 3E). Mice vaccinated with high dose Fluzone mixed with CDA had on average HAI titers of 1:80–1:160 against B/WA/19 (Figure 3E). Mice vaccinated with low dose Fluzone mixed with TRAC478 or CDA had low to no detectable HAI titers. In contrast to Fluzone, mice vaccinated with unadjuvanted Flublok had on average HAI titers ~1:1024 against B/AS/21 (Figure 3F). Mice vaccinated with high dose Flublok with either adjuvant had ~4-fold higher HAI titers than unadjuvanted Flublok vaccinated mice, and mice vaccinated with low dose Flublok with either adjuvant had ~2-fold higher HAI titers against B/Aus/21 than mice vaccinated with unadjuvanted Flublok vaccine (Figure 3F). Similarly to Fluzone vaccinated mice, mice vaccinated with high dose Flublok mixed with either adjuvant had average HAI titers between 1:40 and 1:80 against B/WA/19 (Figure 3F). While mice vaccinated with low dose Flublok plus TRAC478 had low to undetectable HAI titers against B/WA/19, mice vaccinated with low dose Flublok plus CDA had on average HAI titers of 1:40–1:80 against B/WA/19 (Figure 3F).

**Figure 4:**
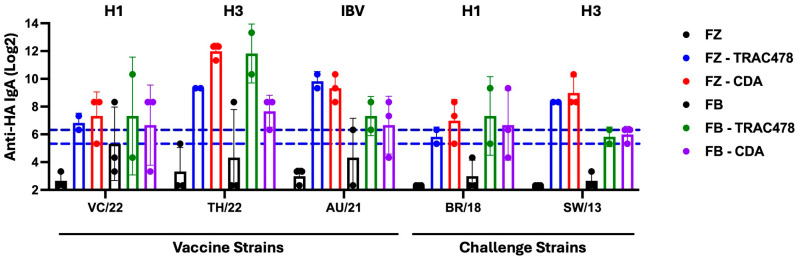
**Anti-HA IgA antibody titers induced by adjuvanted and non-adjuvanted 2024–2025 Fluzone or Flublok seasonal influenza vaccination in bronchial lung lavage.** Anti-HA IgA titers were assessed against the three vaccines strains (VC/22, TH/22, AU/21) and the two challenge viruses (BR/18 and SW/13) B. Log_2_ IgA titers for individual mice are shown as scatter dot plots with bars representing the mean titer of each group and error bars indicating the standard deviation. Dotted lines indicate a 1:40 to 1:80 titer range.

To determine if vaccinated mice had anti-HA specific IgA responses in respiratory mucosal tissues, bronchial lavage fluid was collected and assessed for IgA antibody binding to each HA component of the vaccine (VC/22, TH/22, AU/21), as well as the HA of the two challenge strains (BR18 and SW/13) (Figure 4). Mice vaccinated with Fluzone or Flublok with no adjuvant had low to undetectable IgA titers in the lungs; however, mice vaccinated with these vaccines formulated with TRAC478 or CDA had 4–256 fold increase in anti-HA IgA titers compared to mice vaccinated with unadjuvanted vaccines.

### 3.3. B Cell ELISPOTS

**Figure 5:**
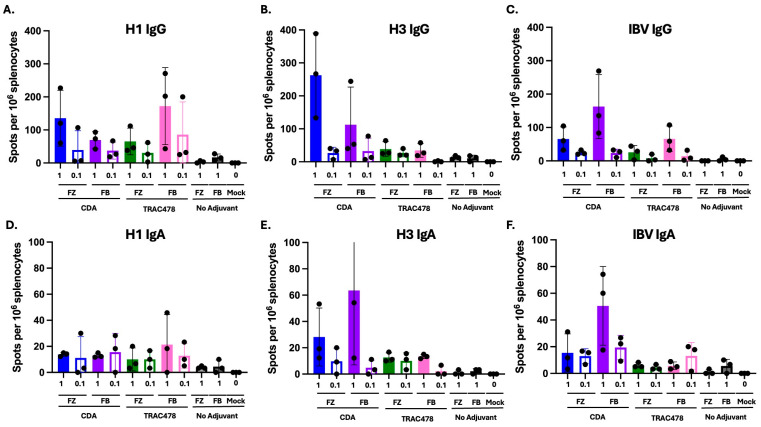
**B cell response after vaccination.** Spleens were collected 7 days after final vaccination for ELISPOT assay to enumerate IgG (**A**–**C**) and IgA (**D**–**F**) antibody secreting cells against individual vaccine components. The number of spots per 1 × 10^6^ splenocytes were calculated for each individual mouse (dots) and the average spots per group (bars) plus/minus the standard error. Statistical analysis was performed using one way ANOVA with multiple comparisons with GraphPad Prism 9 software (GraphPad, San Diego, CA, USA).

Spleens were harvested on day 37 (9 days post-boost to determine the number of ASCs (Figure 5). Mice vaccinated with a high dose of Fluzone or Flublok mixed with CDA or TRAC had higher numbers of HA-specific IgG antibody secreting cells (ASC) against the H1 vaccine component than mice vaccinated with low dose adjuvanted vaccines (Figure 5A). Mice vaccinated with a high dose of Fluzone or Flublok mixed with CDA had higher number of HA-specific IgG antibody secreting cells (ASC) against the H3 vaccine component and similar numbers against the IBV vaccine component than mice vaccinated with any of the other vaccines (Figure 5B,C). These same mice had low to undetectable IgA ASCs against H1 vaccine component regardless of which vaccine they administered (Figure 5D). Mice vaccinated with high dose Flublok plus CDA had the highest average titer of IgA ASCs against the H3 and IBV vaccine components (Figure 5E,F).

### 3.4. T Cell ELISPOTS

**Figure 6:**
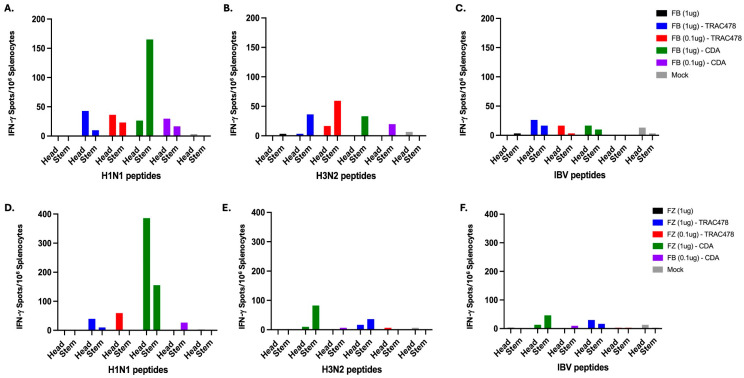
**Cumulative IFN-γ spot counts elicited by adjuvanted COBRA rHA-based vaccines.** Splenocytes collected on day 39 were pooled for each vaccine group and probed for IFN-γ secretion against overlapping 14–15-mer peptide pools derived from the A/New York/18/2009 (H1N1) (**A**,**D**), A/Perth/16/2009 (H3N2) (**B**,**E**) or B/Malaysia/2506/2004 (**C**,**F**) for the head domain and peptide pools for the stem domain of HA. The cumulative number of IFN-γ secreting cells per million splenocytes were assessed by ELISpot. Spot counts were plotted for FB vaccinated mice (**A**–**C**) and FZ vaccinated mice (**D**–**F**) and colored based on formulation of adjuvant. Each mock control group representing animals immunized with vaccines that did not contain rHA (PBS and adjuvant) (gray) were also included in this analysis.

**Figure 7:**
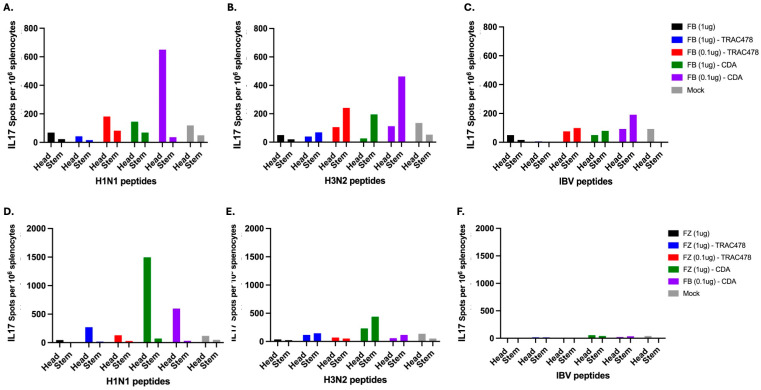
**Cumulative IL-17 spot counts elicited by adjuvanted COBRA rHA-based vaccines.** Splenocytes collected on day 39 were pooled for each vaccine group and probed for IL-17 secretion against overlapping 14–15-mer peptide pools derived from the A/New York/18/2009 (H1N1) (**A**,**D**), A/Perth/16/2009 (H3N2) (**B**,**E**) or B/Malaysia/2506/2004 (**C**,**F**) for the head domain and peptide pools for the stem domain of HA. The cumulative number of IL-17 secreting cells per million splenocytes were assessed by ELISpot. Spot counts were plotted for FB vaccinated mice (**A**–**C**) and FZ vaccinated mice (**D**–**F**) and colored based on formulation of adjuvant. Each mock control group representing animals immunized with vaccines that did not contain rHA (PBS and adjuvant) (gray) were also included in this analysis.

**Figure 8:**
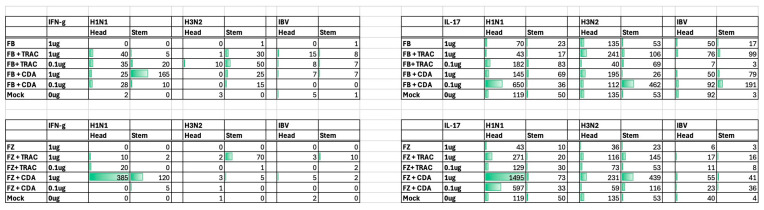
**Representation of the number of spots following peptide stimulation of HA-specific IFN-γ and IL-17 secreting cells per vaccine group.** The number of spots detected following stimulation of splenocytes with peptide representing the head domain of HA or the stem domain of HA for H1, H3, or IBV based upon the spots in Figure 6 and Figure 7. Mice were vaccinated with a high dose or low dose of Flublok (FB) or Fluzone (FZ) formulated with either TRAC478 or CDA, and splenocytes were assessed using ELISpot. The green bars graphically represent the number of spots per vaccine group.

Spleens were collected 9 days following the second vaccination (day 37), homogenized, pooled per each vaccination group, and assessed for H1 and H3 HA-specific interferon-gamma (IFN-γ) (Figure 6) or IL-17 (Figure 7) secreting splenocytes. On average, mice vaccinated with unadjuvanted Fluzone or Flublok had low to undetectable IFN-γ or IL-17 secreting splenocytes against any of the vaccine strains. Mice vaccinated with either vaccine plus TRAC478 or CDA adjuvant had detectable IFN-γ or IL-17 secreting T cell splenocyte responses with the highest responses against the H1 HA component in the vaccine. Graphic representation of the spots is depicted in Figure 8.

### 3.5. Influenza Virus Challenge

**Figure 9:**
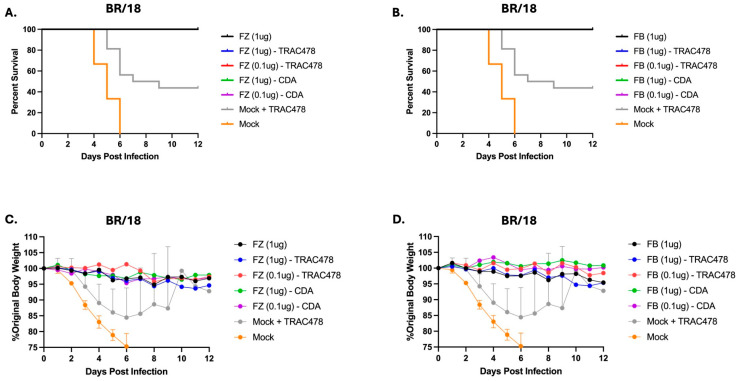
**Survival and weights following challenge with A/Brisbane/02/2018 H1N1 influenza viruses.** Mice were challenged with BR/18 H1N1 virus at 3.6 × 10^4^ PFU/mouse. Mice were monitored for weights and clinical symptoms daily. Percent survival (**A**,**B**) and percent original weight of mice (**C**,**D**) is shown over a 12-day period.

To determine if each vaccine and adjuvant combination could protect against viral infection, vaccinated mice were challenged with influenza virus (Figure 9). All vaccinated mice were protected from an BR/18 H1N1 viral challenge (Figure 9A,B) with little weight loss (Figure 9C,D). TRAC478-only vaccinated mice challenged with BR/18 lost ~15% weight by day 6 post-infection with ~50% of the mice surviving infection. Mock mice all succumbed to infection by day 6 post-challenge (Figure 9).

**Figure 10:**
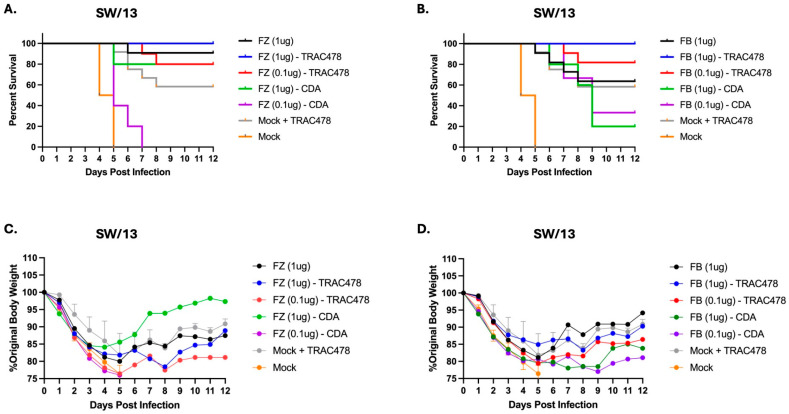
**Survival and weights following challenge with A/Switzerland/9715293/2013 H3N2 influenza viruses.** Mice were challenged with A/Switzerland/9715293/2013 (SW/13) H3N2 virus at 5 × 10^6^ PFU/mouse. Mice were monitored for weight and clinical symptoms daily. Percent survival (**A**,**B**) and percent original weight of mice (**C**,**D**) is shown over a 12-day period with statistical analyses performed using one-way ANOVA.

In contrast to BR/18 infection, mice challenged with SW/13 H3N2 influenza virus all lost weight following challenge regardless of the vaccine used for immunization (Figure 10C,D). On average, mice lost between 15–25% of their original weight between days 4 and 6 post-infection. No mice vaccinated with low dose Fluzone and CDA survived challenge (Figure 10A), whereas 80% of mice vaccinated with low dose Fluzone and mixed with TRAC478 or high dose Fluzone mixed with CDA survived SW/13 challenge. All mice vaccinated with high dose Fluzone and adjuvanted with TRAC478 survived challenge. Similar survival results were observed with mice vaccinated with Flublok with all mice vaccinated with high dose Flublok mixed with TRAC478 surviving SW/13 challenge (Figure 10B).

**Figure 11:**
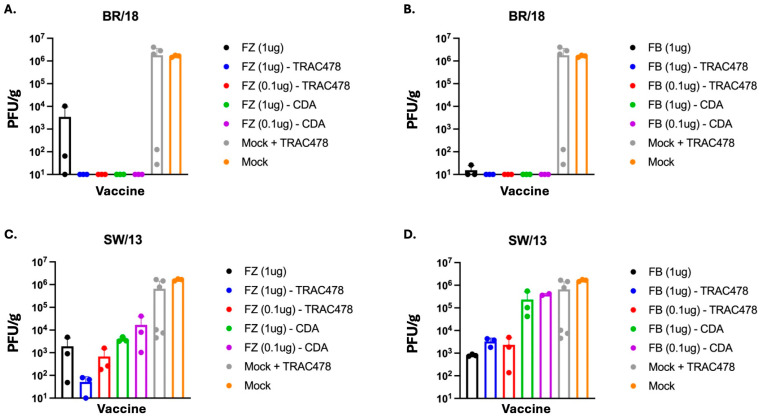
**Viral lung titers.** Viral lung titers were determined by plaque assays from lungs collected from vaccinated mice challenged with BR/18 H1N1 influenza virus (**A**,**B**) or SW/13 H3N2 influenza virus (**C**,**D**) on day 3 post-infection. Each individual mouse lung viral titer is recorded as PFU/g per mouse (dots) and the average viral titer per group (bar).

Mock vaccinated mice or mice vaccinated with TRAC478 only had high BR/18 viral lung titers on day 3 post-challenge with an average titer of 1 × 10^6^ PFU/g (Figure 11A,B). There were low to undetectable viral titers in the lungs of mice vaccinated with Fluzone only, but no detectable viral titers in mice vaccinated with Fluzone mixed with either TRAC478 or CDA (Figure 11A). Mice vaccinated with Flublok mixed with either TRAC478 or CDA had no detectable BR/18 viral lung titers on day 3 post-challenge (Figure 11B). Mock vaccinated mice or mice vaccinated with TRAC478 only challenged with SW/13 also had high viral titers on day 3 post-challenge with an average titer of 7 × 10^5^ PFU/g (Figure 11C,D). Mice vaccinated with unadjuvanted Fluzone had an average titer of 2 × 10^3^ PFU/g, whereas mice vaccinated with high dose Fluzone mixed with TRAC478 had two log lower viral titers (Figure 11C). Mice vaccinated with either high dose or low dose Fluzone mixed with CDA had similar viral titers as mice administered unadjuvanted Fluzone. Mice vaccinated with unadjuvanted Flublok also had ~1 × 10^3^ PFU/g of SW/13 virus on day 3 post-infection that was similar to lung titers from mice vaccinated with Flublok (high or low dose) mixed with TRAC478 (Figure 11D). In contrast, mice vaccinated with Flublok (high or low dose) mixed with CDA had two log higher viral lung titers than mice vaccinated with Flublok mixed with TRAC478 with titers similar to mock vaccinated mice (Figure 11D).

## 4. Discussion

Overall, the goal of this study was to determine whether commercial influenza vaccines, which are intended for intramuscular delivery, can be effectively used for intranasal administration. Fluzone is a split-inactivated influenza virus developed from whole virus generated in embryonated chicken eggs [18]. Flublok contains recombinant HA proteins produced from insect cells [19]. Both vaccines are manufactured by Sanofi Pasteur (Swiftwater, PA, USA) and are updated annually following World Health Organization (WHO) recommendations for inclusion of influenza A and B strains in the vaccine. In most seasons, Fluzone is the primary choice for vaccination of people in the U.S. and Europe [19]. In this study, Fluzone and Flublok were delivered intranasally in mice formulated with or without one of two novel adjuvants, CDA or TRAC478 [15,16,20,21].

Mice were administered both these vaccines intranasally with or without adjuvant containing a high dose (1 μg of each HA) or a low dose (0.1 μg of each HA) of vaccine. Mice vaccinated with a high dose of either vaccine without adjuvant elicited anti-HA antibodies equal to the levels elicited by low dose vaccine plus adjuvant indicating a dose sparing use of vaccine. Mice administered high dose vaccine with adjuvant had significant anti-HA antibody titers compared to mice administered low dose vaccine plus adjuvant, thereby, once again, demonstrating a dose-dependent response following vaccination. These overall IgG anti-HA antibody titers translated into HAI activity with similar adjuvant dependent responses. However, CDA mixed with the high dose of vaccine was more effective at eliciting anti-H3 and IBV HA ASCs than TRAC478 following intranasal administration, but there was no statistical difference in the levels of anti-H1 HA-specific ASCs, regardless of the vaccine dose or adjuvant used in the vaccine. Both TRAC478 and CDA elicited mixed T helper (Th) CD4^+^ T cell responses [22,23,24,25,26,27]. CDA is a bis-(3,5)-cyclic dimeric adenosine monophosphate that is a stimulator of interferon gene (STING) activator and elicits strong mucosal immune responses [12]. This secondary signaling molecule activates the STING protein, resulting in type I IFN and TNF-α (interferon and tumor necrosis factor-alpha) production [28]. Previously, our group demonstrated that CDA elicits both lung mucosal and systemic antibody and T cell responses following intranasal vaccination with recombinant HA and NA proteins [20,21,28]. TRAC478 is a combination of two synthetic toll-like receptor (TLR) agonists and stimulates robust T cell responses [15,16]. Both these synthetic TLR agonists signal through TRIF and MyD88 pathways that ultimately drive the production of pro-inflammatory cytokines for improved T cell priming [29], as well as enhanced humoral responses [29]. TLR7 and TLR8 each primarily recognize ssRNA within the endosome [30]. Combining TLR7 and TLR8 agonists enhances dendritic cell activation and antigen presentation, resulting in a robust Th1 immune response [30,31,32]. Co-encapsulating TLR4 and TLR7/8 within liposomes results in synergistic innate and adaptive immune responses characterized by a balanced Th1/Th2 response and longer-lasting protection [19,20,33,34,35,36].

TLR4 agonists have been administered intranasally with inactivated influenza vaccines [34]. Mice vaccinated with influenza vaccine and a synthetic toll-like receptor 4 agonist, CRX-601, elicited strong local and systemic IgG and IgA antibodies against H3N2 influenza viral proteins and protected mice against two heterologous influenza virus challenges [33]. However, these mice had polyfunctional antigen-specific Th17 cells (CD4^+^IL-17A^+^TNF-α^+^) following intranasal vaccination with increased weight loss and morbidity early in infection but recovered. The vaccine-primed Th17 cells were associated with an infiltration of neutrophils into lung tissues that was partially responsible for mediating the increased morbidity [33]. In this study, using TLR4 and TLR7/8 mixed adjuvants, there was no enhancement of disease following vaccination or challenge. However, following viral infection, there was an increased weight loss and less survival of mice vaccinated with the low dose of Fluzone or Flublok mixed with CDA adjuvant following infection with the H3N2 influenza virus compared to TRAC478-only vaccinated mice challenged with the same virus. Therefore, our data reveals that the choice of adjuvant, particularly CDA, can cause severe detrimental effects following intranasal administration in future trials.

Following challenge with an H1N1 or H3N2 influenza virus, there were little statistical differences in weight loss or survival, regardless of whether the mice were vaccinated with either vaccine with or without adjuvant. Therefore, despite differences in elicited immune responses between vaccine formulations and vaccine doses, mice vaccinated with unadjuvanted Fluzone or Flublok were completely protected against virus challenge. There were few differences in viral lung titers at 3 days following H1N1 influenza virus challenge between vaccine groups; however, mice vaccinated with Fluzone plus CDA had a 2–3 log increase in viral titers compared to mice vaccinated with Flublok only or Flublok plus CDA. Similarly, mice vaccinated with Fluzone plus CDA had 1.5 log higher viral lung titers than mice vaccinated with Fluzone plus TRAC478. Why do immune responses elicited by these influenza vaccines plus TRAC478 control viral infection more effectively than CDA since they act through similar mechanisms? This is curious since Flublok contains three times more HA per vaccine dose (45 μg) than Fluzone (15 μg) [10]. And Flublok injected intramuscularly is more effective against influenza virus infection than other influenza standard-dose vaccines in older adults [10]. Flublok has fewer adverse advents following administration than other influenza vaccines and is now approved for children 9 years and older as well as pregnant women [10].

There may also be differences in the elicitation of memory B cells (MBCs) following vaccination. Humans vaccinated with Flublok had robust HAI and HA-specific MBC responses with increased frequencies of switched B cell memory and IgG memory against all three vaccine HA components (H1, H3, and IBV) compared to Fluzone. In addition, Flublok-vaccinated participants also had increased IgA resting memory and IgG activated memory cells against the H1 and H3 vaccine HA components, and IgG resting memory against the H1 and IBV HA components. People vaccinated with Fluzone had robust, but comparatively lower HA specific MBC responses that were characterized by increased IgG memory cells against the H1 and IBV HA, but reduced switched memory cells compared to Flublok vaccinated people [34]. Since the participants in these studies were administered the vaccine intramuscularly, it is not clear if these same effects would be observed following intranasal delivery and future studies in people are needed.

The addition of adjuvants to Fluzone was also effective at stimulating serum immune responses following intranasal vaccination [37]. After a single intranasal immunization, the adjuvanted influenza vaccine elicited elevated serum HAI titers against the three HA antigens present in the vaccine [37] with seroconversion rates between 67 and 100% against each vaccine strain with significant HAI titers against five additional H3N2 influenza drifted variants and completely protected ferrets against influenza virus challenge [37]. A significant number of people do not take the annual seasonal influenza vaccine because of needle hesitancy [38,39,40] and would prefer an oral- or nasal-delivered vaccine. FluMist is a licensed intranasal influenza vaccine based upon a live attenuated influenza virus (LAIV) [41]; however, this vaccine is approved for individuals between the ages of 2 and 49 years [42]. The possibility of repurposing spilt-inactivated or recombinant HA commercially available vaccines for intranasal delivery could increase uptake for all age groups and effectively vaccinate more people per season.

## 5. Conclusions

Commercial vaccines that are traditionally given intramuscularly were administered intranasally in mice using two different adjuvants. Intranasal vaccinations of adjuvanted Fluzone or Flublok elicited higher antibody response compared to unadjuvanted vaccinations. The vaccines adjuvanted with TRAC478 elicited antibodies that protected vaccinated mice against H1N1 and H3N2 viruses compared to mice vaccinated with vaccine mixed with the CDA adjuvant. Repurposing commercial intramuscular vaccines to an intranasal platform may be an effective delivery method to vaccinate hosts to the site of infection.

## Data Availability

Data can be provided upon request from the corresponding author.
